# The effects of 405 nm light on bacterial membrane integrity determined by salt and bile tolerance assays, leakage of UV-absorbing material and SYTOX green labelling

**DOI:** 10.1099/mic.0.000350

**Published:** 2016-09

**Authors:** Karen McKenzie, Michelle Maclean, M. Helen Grant, Praveen Ramakrishnan, Scott J. MacGregor, John G. Anderson

**Affiliations:** ^1^​ Robertson Trust Laboratory for Electronic Sterilisation Technologies (ROLEST), University of Strathclyde, 204 George Street, Glasgow, Scotland G1 1XW, UK; ^2^​ Department of Biomedical Engineering, University of Strathclyde, Wolfson Centre, 106 Rottenrow, Glasgow, Scotland G4 0NW, UK

**Keywords:** bacteria, membrane damage, cell leakage, 405 nm light, inactivation

## Abstract

Bacterial inactivation by 405 nm light is accredited to the photoexcitation of intracellular porphyrin molecules resulting in energy transfer and the generation of reactive oxygen species that impart cellular oxidative damage. The specific mechanism of cellular damage, however, is not fully understood. Previous work has suggested that destruction of nucleic acids may be responsible for inactivation; however, microscopic imaging has suggested membrane damage as a major constituent of cellular inactivation. This study investigates the membrane integrity of *Escherichia coli* and *Staphylococcus aureus* exposed to 405 nm light. Results indicated membrane damage to both species, with loss of salt and bile tolerance by *S. aureus* and *E. coli*, respectively, consistent with reduced membrane integrity. Increased nucleic acid release was also demonstrated in 405 nm light-exposed cells, with up to 50 % increase in DNA concentration into the extracellular media in the case of both organisms. SYTOX green fluorometric analysis, however, demonstrated contradictory results between the two test species. With *E. coli*, increasing permeation of SYTOX green was observed following increased exposure, with >500 % increase in fluorescence, whereas no increase was observed with *S. aureus*. Overall, this study has provided good evidence that 405 nm light exposure causes loss of bacterial membrane integrity in *E. coli*, but the results with *S. aureus* are more difficult to explain. Further work is required to gain greater understanding of the inactivation mechanism in different bacterial species, as there are likely to be other targets within the cell that are also impaired by the oxidative damage from photo-generated reactive oxygen species.

## Introduction

The antimicrobial action of violet-blue 405 nm light has been increasingly reported over the last decade, with numerous research articles highlighting the potential of this novel light technology for decontamination and infection control applications (Maclean *et al.*, 2013, 2015; McKenzie *et al.*, 2014; Dai *et al.*, 2013a, b; Bache *et al.*, 2012). Visible 405 nm light has provided a safer alternative to traditional UV light decontamination technologies, where there is significant risk to human health upon continued exposure (Bolton & Linden, 2003). Subsequently, the use of 405 nm light has generated increasing interest for both clinical and food decontamination-related applications (Maclean *et al.*, 2015; Dancer, 2014; Bache *et al.*, 2012; Luksiene, 2009).

The wide antimicrobial action of violet-blue light in the region of 405 nm for inactivation of micro-organisms in suspension, on surfaces and within biofilms has been demonstrated (McKenzie *et al.*, 2013, 2014; Maclean *et al.*, 2009, 2013; Murdoch *et al.*, 2012; Endarko *et al.*, 2012; Dai *et al.*, 2012; Enwemeka *et al.*, 2008; Guffey & Wilborn, 2006). The use of violet-blue light for clinical applications has also been investigated, including its potential for wound decontamination (Zhang *et al.*, 2014; Dai *et al.*, 2013a, b; McDonald *et al.*, 2011) and for environmental decontamination applications (Maclean *et al.*, 2010, 2015; Bache *et al.*, 2012).

The antimicrobial inactivation mechanism of 405 nm light has been accredited to the photoexcitation of intracellular photosensitive porphyrin molecules, which subsequently results in the production of reactive oxygen species (ROS), which induces non-specific oxidative damage and cell death (Dai *et al.*, 2012; Maclean *et al.*, 2008; Hamblin & Hasan, 2004). However, despite increasing interest in the antimicrobial properties of violet-blue 405 nm light, investigation into the specific mode of action has generated only limited results. Previous work had hypothesized that exposure to violet-blue light may cause DNA damage similar to that of UV light (Enwemeka *et al.*, 2008); however, more recently, membrane damage has been indicated as having a role in microbial inactivation, with membrane degradation of *Pseudomonas aeruginosa* evidenced using transmission electron microscopy (Dai *et al.*, 2013b). Evidence in support of this hypothesis is limited, and further confirmation of the mechanism of inactivation is required.

The aim of the current study was to investigate the effect of 405 nm light on bacterial cell membrane integrity. *Escherichia coli* and *Staphylococcus aureus* were selected as key model organisms, representing Gram-negative and Gram-positive bacterial species. Assessment of membrane damage was investigated by multiple techniques including selective and non-selective plating for assessing loss of tolerance to specific environmental stress factors, as well as spectrophotometry to assess leakage of nucleic acid materials and uptake of SYTOX green dye through permeated membranes.

## Methods

### Bacterial preparation.

*E. coli* NCTC 9001 and *S. aureus* NCTC 4135 (National Collection of Type Cultures) were cultured in nutrient broth (Oxoid) at 37 °C for 18–24 h, under rotary conditions (120 rpm). Post-incubation, cultures were centrifuged at 3939 ***g*** for 10 min, and the pellet was re-suspended and diluted, if required, in PBS (Oxoid) for experimental use.

### 405 nm light exposure of bacterial suspensions.

The light source used in this study was a light-emitting diode (LED) array (ENFIS PhotonStar Innovate UNO 24, PhotonStar Technologies), powered by a 40 V Phillips Xitanium LED driver, with peak output at approximately 405 nm and a bandwidth of 15 nm at full width half-maximum. Arrays were attached to a heat sink and fan, to improve thermal management and minimize heat transfer to exposed samples. Irradiance was measured using a radiant power meter and photodiode detector calibrated at 405 nm (LOT Oriel).

Sample volumes of 3 ml were positioned directly below the LED array, at a distance of 5 cm, giving an irradiance of approximately 65 mW cm^−2^ at the sample surface. These settings were selected as they were optimal for achieving a uniform irradiance distribution across the sample surface. Samples were exposed to increasing applied doses of 405 nm light, using exposure times of up to 180 min, with dose calculated as irradiance (W cm^−2^)×exposure time(s). Identical samples, exposed to normal laboratory lighting (approximately 0.06 mW cm^−2^), were set up as experimental controls. Temperature of the light-exposed and control samples was monitored using a thermocouple (Kane May KM340), and a maximum temperature increase of 2 to 3 °C occurred in samples that received the greatest light dose thereby verifying that the inactivation was not due to a heating effect from the light source.

### Determination of sub-lethal injury by loss of salt and bile tolerance.

Lethal and sub-lethal injury resulting from 405 nm light treatment was determined using differential plating methods. Sub-lethally damaged or injured bacteria are less likely to grow on selective media due to the harsher environmental growth conditions than on non-selective media. Therefore, this principle can be used to estimate the level of sub-lethal damage within a bacterial population (Carson *et al.*, 2002). To do this, we treated population densities of 10^7 ^c.f.u. ml^−1^ with 405 nm light, and post-exposure samples were plated onto both non-selective [nutrient agar (NA; Oxoid)] and selective media [mannitol salt agar (MSA; Oxoid) and violet-red bile agar (VRBA; Oxoid), for *S. aureus* and *E. coli*, respectively]. MSA and VRBA were selected for use due their high salt, NaCl (7.5 %) and bile (1.5 %) concentrations. *S. aureus* and *E. coli* are highly tolerant to these respective conditions. However, upon injury, loss of tolerance to these conditions is indicative of membrane damage; therefore, this method was selected as an initial indicator of membrane damage. Plates were incubated at 37 °C for 18 to 24 h then enumerated, with results recorded as c.f.u. ml^−1^.

### Leakage of UV-absorbing materials.

Quantification of the leakage of nucleic acid material from the bacterial cells was used as an indicator of membrane damage. We exposed 9-log_10_ population densities to 405 nm light treatment, and post-exposure samples were centrifuged at 3939 ***g*** for 5 min and the supernatant was extracted and analysed using a Bio-mate 5-UV-Vis spectrophotometer (Thermo-Scientific). The absorbance of the supernatant at 260 nm was measured to indicate the presence of leaked nucleic acids (Ukuku *et al.*, 2013; Carson *et al.*, 2002). Results were compared to those of non-exposed control samples.

### Fluorescence labelling with SYTOX green.

To further indicate membrane damage resulting from 405 nm light exposure, we stained cells with SYTOX green (Life Technologies), a high-affinity nucleic acid stain that can only permeate cells with compromised plasma membranes. For this technique, light-exposed samples, at a density of 10^9 ^c.f.u. ml^−1^, were centrifuged (as previously described) and cell pellets were immediately re-suspended in 100 µl 5 mM SYTOX green solution and incubated in the dark for 20 min.

### Fluorescence detection methods.

The degree of SYTOX green binding to bacterial DNA was measured using a fluorescence spectrophotometer (Shimadzu RF5301). Excitation was at 490 nm, and emission spectra were recorded between 500 and 700 nm, with peak fluorescence detected at 523 nm. Fluorescence intensity was expressed as a percentage increase over non-exposed control samples. For visualization of fluorescently stained bacteria, samples were seeded on glass coverslips (13 mm) and viewed under a Zeiss AxioImager Z1 fluorescent microscope for green fluorescence using an FITC filter (filter set 10) and a 100× oil lens (NA=0.5).

### Statistical analysis.

Experimental data are an average of a minimum of triplicate independent experimental results, measured in duplicate (*n*≥6). sem was calculated for all data. Data were analysed using one-way ANOVA test with Minitab 15 statistical software, with significant differences accepted at *P*≤0.05.

## Results

### Establishment of bacterial inactivation kinetics

**Fig. 1.**
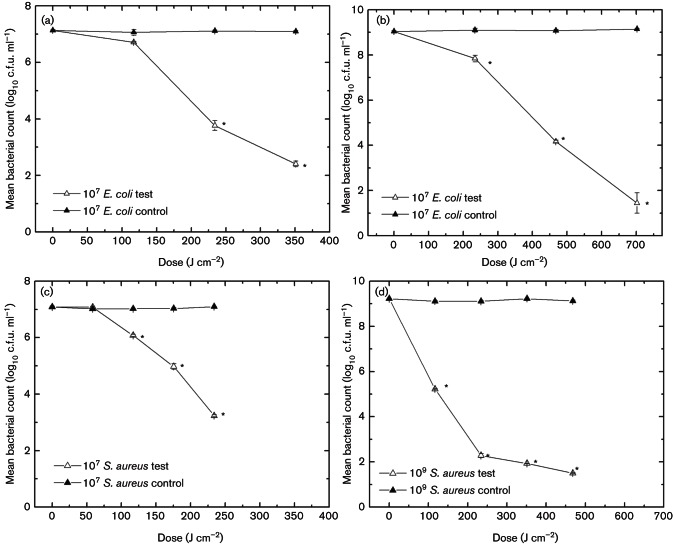
Inactivation kinetics of *E. coli* and *S. aureus* with exposure to 405 nm light, with an irradiance of 65 mW cm^−2^. (a and b) *E. coli* at 10^7^ and 10^9 ^c.f.u. ml^−1^, respectively; (c and d) *S. aureus* at 10^7^ and 10^9 ^c.f.u. ml^−1^, respectively. Non-exposed control samples showed no significant change. An asterisk (*) represents significant bacterial inactivation, when compared to associated non-exposed control (*P*≤0.05). Each data point is a mean value±sem (*n*≥6).

The inactivation kinetics of *E. coli* and *S. aureus*, at population densities of 10^9^ and 10^7 ^c.f.u. ml^−1^, as a function of dose are shown in Fig. 1. At both population densities, *S. aureus* is shown to be more susceptible to 405 nm light than *E. coli*. Exposure of 10^7 ^c.f.u. ml^−1^ population at 117 J cm^−2^ highlighted significant reduction of *S. aureus* (1-log_10_) (*P*≤0.05), compared to only 0.4-log_10_ reduction observed with *E. coli*. Similarly following an applied dose of 234 J cm^−2^, a 3.8-log_10_ reduction was recorded for *S. aureus* compared to a 3.3-log_10 _decrease in *E. coli* population. This difference in susceptibility is more apparent at the higher population density of 10^9 ^c.f.u. ml^−1^ (Fig. 1b, d). *S. aureus* demonstrated a 7.7-log_10_ reduction following 468 J cm^−2^, whereas *E. coli* required exposure to 702 J cm^−2^ to achieve the same level of inactivation. It would be expected that for inactivation of higher bacterial populations, greater exposure to 405 nm light would be required and that is indeed the case for *E. coli*. The results for *S. aureus* do not appear to show this trend, with greater initial susceptibility at the higher population density observed. This may be a result of there being a greater density of cells available for the 405 nm photons to interact with, and this being more apparent with the *S. aureus* (compared to the *E. coli*) due to the greater overall sensitivity of this organism to 405 nm light.

These kinetics were used as baseline curves for comparison in the subsequent microbial and biochemical assays, with the population density used being determined by the specific protocol.

### Determination of sub-lethal injury by loss of salt and bile tolerance

Evidence of sub-lethal injury was ascertained by plating the light-exposed bacterial samples onto both NA medium (non-selective) and MSA and VRBA media (selective for *S. aureus* and *E. coli*, respectively), with sub-lethally damaged populations quantified from the difference in counts between the growth on the selective versus non-selective media.

**Fig. 2.**
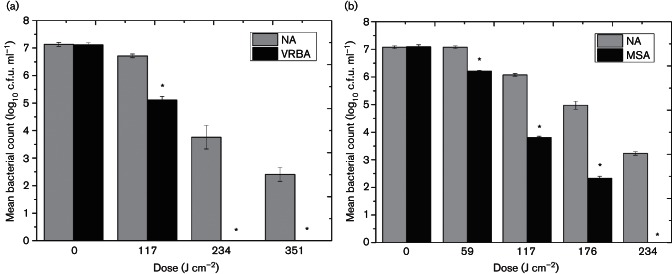
Demonstration of lethal and sub-lethal damage of (a) *E. coli* and (b) *S. aureus* exposed to 405 nm light by comparison of post-exposure survivors plated on non-selective and selective media. Bacterial populations were exposed to an irradiance of 65 mW cm^−2^. VRBA and MSA were selected for use due to their high bile and salt concentrations, respectively, to provide selective pressures which discourage growth of bacteria with reduced membrane integrity. NA was used as the non-selective growth medium. An asterisk (*) represents significant bacterial inactivation on selective agar, when compared to associated non-selective counts (*P*≤0.05). Each data point is a mean value±sem (*n*≥6).

Significant differences between VRBA (1.5 % bile salts) and NA *E. coli* counts were demonstrated (Fig. 2a), where following only 117 J cm^−2^, a statistically significant sub-lethally damaged population (1.6-log_10_) was observed (*P*≤0.05). Results further highlight that following 234 and 351 J cm^−2^, the entire bacterial population demonstrates sub-lethal damage, as shown by the complete inhibition of bacterial growth on VRBA.

Similarly, results highlight that, for *S. aureus*, following an applied dose of 59 J cm^−2^, significant differences (*P*=0.002) in bacterial counts were observed between samples plated onto MSA (7.5 % NaCl) and NA (Fig. 2b). Each subsequent dose demonstrated increasing inhibition of cell growth in both media, with differences in bacterial counts obtained after plating on selective and non-selective media, of between 0.9-log_10_ and 3.2-log_10_ being recorded.

### Leakage of UV-absorbing material

**Fig. 3.**
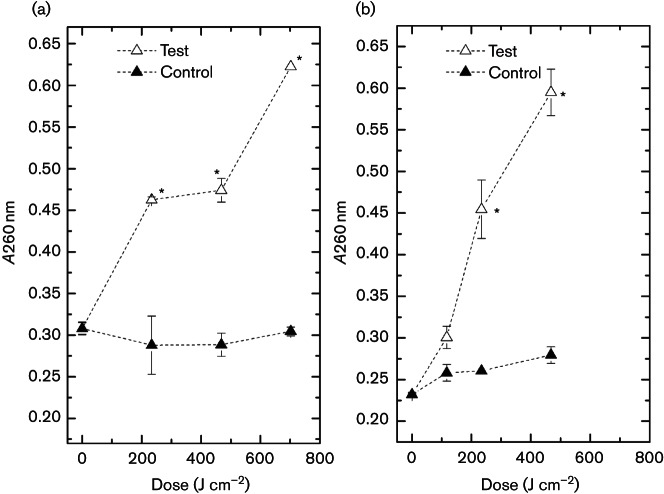
Absorbance measurements of (a) *E. coli* and (b) *S. aureus* cell supernatants at 260 nm following 405 nm light exposure (65 mW cm^−2^). An asterisk (*) represents a significant increase in absorbance reading when compared to the equivalent non-exposed control samples (*P*≤0.05). Each data point is a mean value±sem (*n*≥3).

Leakage of UV-absorbing material from exposed bacterial cells was measured at increasing dose levels, with the doses used determined by the inactivation kinetics (Fig. 1a, b). Results in Fig. 3 highlight that for both *E. coli* and *S. aureus*, upward trends in absorbance at 260 nm were observed with increasing light exposure. Absorbance readings from control samples did not differ significantly over the entire exposure period. Results presented in Fig. 3(a) demonstrate an increase in absorbance at 260 nm for *E. coli* following a dose of 234 J cm^−2 ^(*P*≤0.05), with absorbance readings continuing to increase following 469 and 702 J cm^−2^. Similarly, results shown in Fig. 3(b) highlight an increase in absorbance at 260 nm for *S. aureus* following 117, 234 J cm^−2^ and almost a threefold increase in absorbance levels following 468 J cm^−2^.

### Identification of bacterial cell membrane damage by SYTOX green labelling

**Fig. 4.**
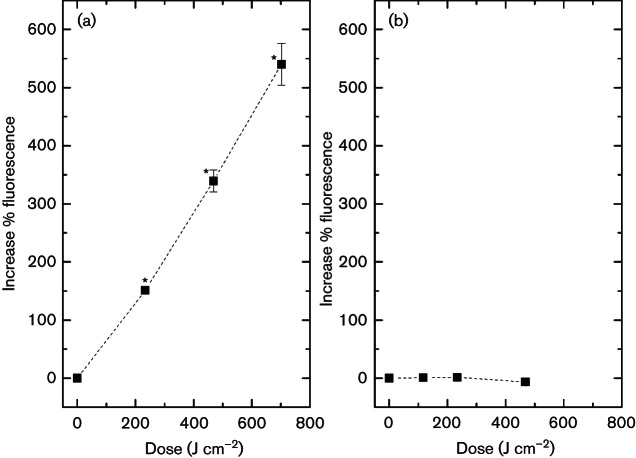
SYTOX green fluorescence at 523 nm of (a) *E. coli* and (b) *S. aureus* cells following increasing doses of 405 nm light exposure (65 mW cm^−2^), using an excitation wavelength of 490 nm. Results are measured as the percentage increase compared to non-exposed control samples. An asterisk (*) represents a significant increase in fluorescence when compared to non-exposed control samples (*P*≤0.05). Each data point is a mean value±sem (*n*≥3).

Results demonstrate a significant enhancement of the fluorescence signal of SYTOX green in exposed *E. coli* cells (Fig. 4a), indicating that membrane integrity was compromised, allowing SYTOX green to enter and attach to nucleic acids. After an applied dose of 234 J cm^−2^, fluorescence had significantly increased by 150 % (*P*=0.001). Exposure to increased doses continued to result in greater fluorescence, with 540 % increase following 702 J cm^−2^ 405 nm light treatment. Interestingly, a similar trend was not observed with light-exposed *S. aureus*, with results (Fig. 4b) demonstrating no significant change in fluorescence signal of SYTOX green, irrespective of the exposure period (*P*≥0.05).

**Fig. 5.**
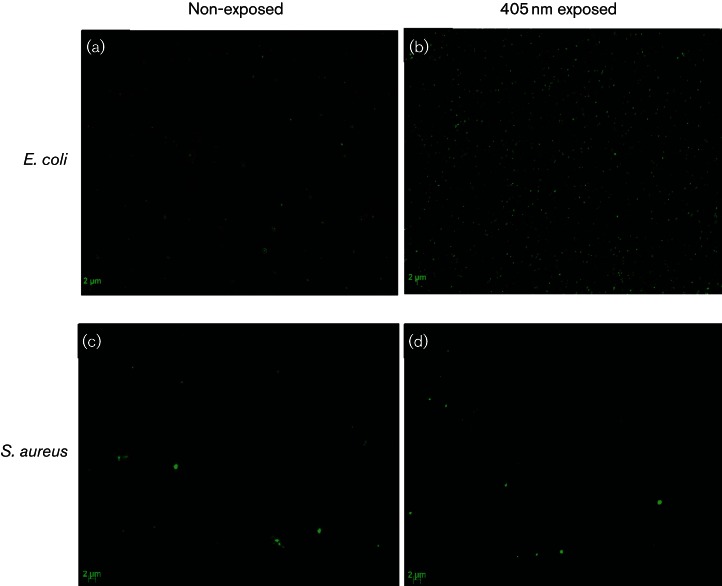
Fluorescence microscopy of SYTOX green stain labelled bacteria. Cells were exposed to a dose of 234 and 117 J cm^−2^, for *E. coli* and *S. aureus*, respectively (first data point in inactivation curve Fig. 1). Cells were stained with 5 mM SYTOX green and brightness in fluorescence between exposed and non-exposed samples was compared visually using an epifluorescent microscope. Images (a) and (b) represent MosaiX images (8×8) of non-exposed and 405 nm exposed *E. coli*. Images (c) and (d) represent non-exposed and 405 nm exposed *S. aureus*. Excitation and emission were 490 nm and >520 nm, respectively, for all samples.

To provide visual evidence of membrane damage by SYTOX green labelling, we viewed bacteria using an epifluorescent microscope. Images of *E. coli* (Fig. 5b) demonstrate a visible increase in green fluorescence following exposure to 234 J cm^−2^ 405 nm light, compared to the non-exposed control (Fig. 5a). These results indicate binding of SYTOX green to nucleic acids, providing evidence of permeation to the membrane structure following 405 nm light exposure. Very slight fluorescence signal was demonstrated by *S. aureus*, but this was negligible compared to the non-exposed cells, thus correlating with the lack of fluorescence signal detected spectrophotometrically (Fig. 4b).

## Discussion

The results generated in this study provide clear evidence that 405 nm light induces bacterial cell membrane damage to *E. coli* but the results with *S. aureus* are less conclusive and require further investigation to fully understand the inactivation mechanism in this organism. In order to further utilize 405 nm light as a decontamination technology, greater understanding of its mechanism(s) of action is required. Furthermore, a better perception of its mode of action may also help elucidate differences in susceptibility between various species, again an area which, to date is speculative, with theories being accredited to differences in bacterial structure and intracellular porphyrin concentrations (Nitzan *et al.*, 2004; Demidova & Hamblin, 2004; Malik *et al.*, 1992).

Results demonstrate that, with increased doses of 405 nm light, greater bacterial inactivation is achieved (Fig. 1). Also, results shown in Fig. 2 demonstrate that 405 nm light can successfully induce both lethal and sub-lethal damage. Loss of salt and bile tolerance by *S. aureus* and *E. coli*, respectively, is indicative of structural damage, more specifically this has been previously ascribed to membrane damage (Carson *et al.*, 2002). Results highlight that, for both bacteria, after only 59 and 117 J cm^−2^ for *S. aureus* and *E. coli*, respectively, no significant lethal inactivation was achieved (less than 0.4-log_10_ reduction). However, a significant degree of sub-lethal damage was induced (1-log_10 _to 1.5-log_10_ reduction) and was shown to increase at a substantially greater rate than complete inactivation levels. These data indicate that even low doses of 405 nm light exposure can initiate damage and that membrane damage may be a major contributor to cellular inactivation. This hypothesis is supported by the visual data provided by Dai and colleagues showing transmission electron microscopy of membrane degradation following visible violet-blue light treatment at 415 nm (Dai *et al.*, 2013b). Sub-lethal injury of microbial cell membranes may alter their permeability and affect their ability to regulate the intracellular environment of the cell sufficiently and/or to expel toxic materials – including ROS (Gilbert, 1984). It is, therefore, possible that 405 nm light can successfully induce membrane damage, which first promotes sub-lethal effects, followed most likely by leakage of cellular components and then by complete lysis of the cell, resulting in cell death. Leakage of cytoplasmic material from the cell was investigated to provide further evidence of potential membrane damage. Numerous studies investigating antimicrobial mechanisms and membrane integrity have highlighted the loss of 260 nm absorbing material as a clear indicator of cytoplasmic membrane damage (Carson *et al.*, 2002; Hugo & Longworth, 1964).

Results shown in Fig. 3, for both *E. coli* and *S. aureus* exposed to 405 nm light, correlate well with the inactivation kinetics shown in Fig. 1, highlighting that, as expected, exposure to increasing doses of 405 nm light results in increased absorbance readings at 260 nm, indicating increasing release of nucleic acids from light-damaged cells. Further spectrophotometric readings highlighted an increase in DNA concentration released from bacterial cells into the extracellular medium following 405 nm light treatment, providing further evidence of light-induced cellular damage. Again results correlate well with the inactivation kinetics (Fig. 1), where release of DNA into extracellular media increases as the bacterial population decreases.

To further investigate the importance of a loss in membrane integrity, we employed fluorescent spectrophotometric analysis and fluorescent microscopy. The results with *E. coli* demonstrated, for the first time to our knowledge, quantitative evidence of the bacterial cell membrane damage following 405 nm light exposure. A number of fluorescence-based assays can be used for detection of cell viability; however, SYTOX green was selected due to its high fluorescence properties and low cellular toxicity (Tashyreva *et al.*, 2013; Roth *et al.*, 1997). Previous studies have demonstrated the use of SYTOX green for assessment of bacterial membrane viability following antimicrobial treatment, where it is utilized as a fluorescent probe for measuring membrane integrity, by diffusing only through damaged cell membranes to bind to intact DNA, resulting in an intense green fluorescence (Breeuwer & Abee, 2000; Roth *et al.*, 1997).

Results from the SYTOX green labelling demonstrated a linear increase in fluorescence with increased 405 nm light exposure for *E. coli*: a 1-log_10_, 5-log_10_ and 8-log_10_ reduction in bacterial counts (data presented in Fig. 1) resulted 151 %, 340 % and 540 % increase in fluorescence of *E. coli*. This increase in fluorescence was also observed visually by microscopy. In comparison, no detectable increase in fluorescence was observed spectrofluorimetrically or microscopically with *S. aureus* following 405 nm light treatment.

Although results of the selective plating and absorbance assays indicated similar trends in cellular damage in both *E. coli* and *S. aureus*, the SYTOX green labelling results highlight that there may be differences in how this damage is imparted in the differing structures of Gram-negative and Gram-positive bacteria. A recent study by Ramakrishnan *et al*. (2016) has provided evidence that the cytotoxic mechanism of 405 nm light in bacteria cells is due to oxidative stress involving predominantly H_2_O_2_ generation, with other ROS contributing to the damage. This oxidative stress is therefore likely to be not only inducing non-specific indirect damage to the cell membrane but also simultaneously targeting various organelles, causing oxidation of proteins responsible for ATP generation and altering the structural integrity of the nucleic acids (Demidova & Hamblin, 2004). A number of limitations for this fluorescent technique have been discussed in previous literature (Lebaron *et al.*, 1998), whereby loss of membrane integrity through antimicrobial treatment may in fact simultaneously and/or sequentially lead to DNA degradation, which will undoubtedly affect viability of the assay (Lebaron *et al.*, 1998; Weichart *et al*, 1997). The reason why SYTOX green fluorescence is not generated to the same extent inside *S. aureus* as in *E. coli* remains unclear; however, such Gram-positive cells have been found to be generally more susceptible to 405 nm light than Gram-negative cells (Fig. 1) (McKenzie *et al.*, 2014; Murdoch *et al.*, 2012; Maclean *et al.*, 2009), and as a result, more rapid oxidative damage to DNA may be occurring. This degradative damage may account for the reduced levels of fluorescence signal from SYTOX green in *S. aureus* suspensions, as SYTOX green binds only to intact DNA helices. It may be that where cells are structurally more complex, DNA degradation may occur only after significant loss of membrane integrity, whereas simultaneous damage may occur with less structurally complex cells. Alternatively DNA repair mechanisms may vary between species, an example being the production of Dps by *E. coli*, a stationary-phase-specific protein that protects against oxidative damage of DNA by exogenous agents (Nair *et al.*, 2004; Demidova & Hamblin, 2004). Future work could investigate the potential for indirect DNA damage by measuring the deoxyguanosine content of exposed cells. Due to its low ionization potential, and thus high susceptiblity to oxidation, it may provide a good marker for analysis to ROS damage to DNA (Pelle *et al.*, 2003).

Another significant difference between *S. aureus* and *E. coli* is that the former species produces the yellow pigment staphyloxanthin (Pelz *et al.*, 2005), which is a membrane-bound carotenoid thought to play some role in protection against oxidative stress and may be a virulence factor enabling detoxification of host immune-system-generated ROS (Sakai *et al.*, 2012). Although staphyloxanthin, which is an antioxidant, is thought to primarily affect membrane lipids, it may also interact with proteins and DNA (Clauditz *et al.*, 2006), and it is possible that the presence and effects of staphyloxanthin may play some role in the differential results observed between the two species in the current study.

In summary, this study has demonstrated for the first time to our knowledge substantial quantitative evidence of the membrane damage evident in *E. coli* bacteria upon exposure to 405 nm light. The results with *E. coli* have demonstrated membrane damage both by biochemical analysis and microscopic fluorescence imaging, whereby leakage of intracellular components and passage of fluorescent dyes have provided significant evidence of permeation of the cell membrane. The results obtained with the Gram-positive *S. aureus* are, however, more difficult to interpret. Data from biochemical analysis of leakage of intracellular components in response to light exposure were similar to those obtained with *E. coli*, but the results using SYTOX staining were different. Consequently, until this discrepancy is resolved, it is not possible to conclude that the results observed with *S. aureus* can be fully ascribed to light-induced cell membrane damage. It is possible that 405 nm light exposure may induce damage to multiple sites within the cell and further work is still required to fully understand the mechanism involved during 405 nm light inactivation.
